# Roles of Long Non-coding RNAs in the Development of Aging-Related Neurodegenerative Diseases

**DOI:** 10.3389/fnmol.2022.844193

**Published:** 2022-03-14

**Authors:** Yu-Qing Ni, Hui Xu, You-Shuo Liu

**Affiliations:** ^1^Department of Geriatrics, The Second Xiangya Hospital of Central South University, Changsha, China; ^2^Institute of Aging and Age-Related Disease Research, Central South University, Changsha, China

**Keywords:** long non-coding RNAs, ageing, neurodegenerative diseases, Alzheimer’s disease, Parkinson’s disease, Huntington’s disease, amyotrophic lateral sclerosis

## Abstract

Aging-related neurodegenerative diseases, including Alzheimer’s disease (AD), Parkinson’s disease (PD), Huntington’s disease (HD), and amyotrophic lateral sclerosis (ALS), are gradually becoming the primary burden of society and cause significant health-care concerns. Aging is a critical independent risk factor for neurodegenerative diseases. The pathological alterations of neurodegenerative diseases are tightly associated with mitochondrial dysfunction, inflammation, and oxidative stress, which in turn stimulates the further progression of neurodegenerative diseases. Given the potential research value, lncRNAs have attracted considerable attention. LncRNAs play complex and dynamic roles in multiple signal transduction axis of neurodegeneration. Emerging evidence indicates that lncRNAs exert crucial regulatory effects in the initiation and development of aging-related neurodegenerative diseases. This review compiles the underlying pathological mechanisms of aging and related neurodegenerative diseases. Besides, we discuss the roles of lncRNAs in aging. In addition, the crosstalk and network of lncRNAs in neurodegenerative diseases are also explored.

## Introduction

Neurodegenerative diseases are a range of disorders characterized by irreversible neuron or myelin sheath loss and gliosis, which deteriorate over time and result in dysfunction. Aging significantly enhances the risk of developing neurodegenerative diseases (Hou et al., 2019). The world population over the age of 60 is expected to double to 22% by 2050. An increase in morbidity and mortality has been noted among the aging individuals (Divo et al., 2018). With aging populations, aging-related neurodegenerative disorders, including Alzheimer’s disease (AD), Parkinson’s disease (PD), Huntington’s disease (HD), and amyotrophic lateral sclerosis (ALS), are prevalent worldwide. Therefore, it is important to gain a deeper insight into biological mechanisms underlying these diseases.

Although the function of protein aggregation in neurodegenerative diseases have received considerable attention, increasing evidence also focus on RNAs as contributing factors in these diseases. Because proteins are vital functional expression of genetic code, messenger RNAs (mRNAs) have been explored more intensively than non-coding RNAs (ncRNAs). Nevertheless, extensive research into the function of ncRNAs has broadened our understanding of diverse biological and pathological processes over the past few decades. Among the subtypes of ncRNAs, long non-coding RNAs (lncRNAs) account for a large proportion, which are a momentous source of molecular regulatory factors in eukaryotic nuclei and are involved in modulating gene expression, including chromatin structure, transcription, and translation (Xiang et al., 2014). LncRNAs are increasingly regarded as indispensable molecules in diverse cellular processes such as differentiation, proliferation, apoptosis and senescence (Mercer and Mattick, 2013; McHugh et al., 2015). Moreover, it has been shown that lncRNAs are associated with various pathological processes of aging-associated diseases, such as neurodegeneration, metabolic imbalances, and cancer (Greco et al., 2015). This review summarizes the pathological mechanisms of aging-related neurodegenerative diseases, including AD, PD, HD, and ALS. Then, we mainly concentrate on the functions of lncRNAs in the progression of these neurodegenerative diseases.

## The Pathological Mechanisms of Aging-Related Neurodegenerative Diseases

**FIGURE 1:**
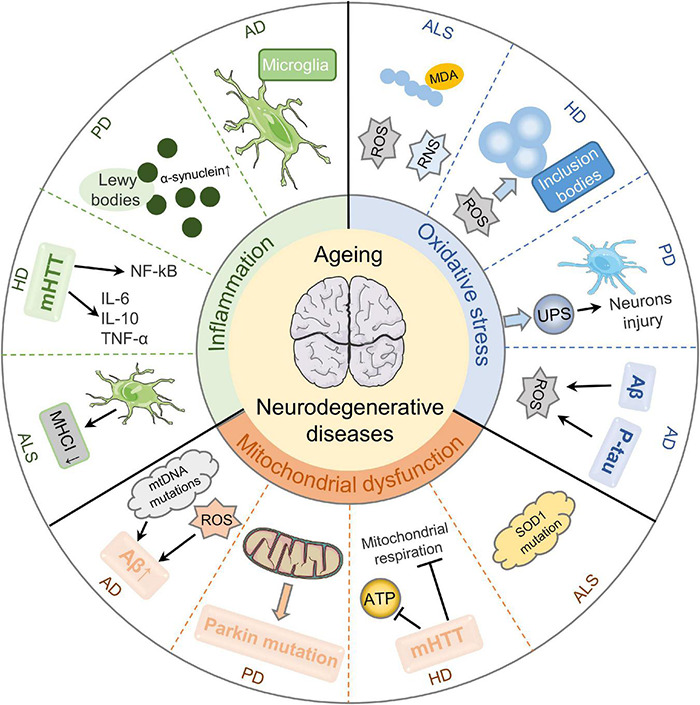
Pathological mechanisms of aging-related neurodegenerative diseases. Mitochondrial dysfunction, oxidative stress, and inflammation are essential cellular and molecular events in the pathogenesis of aging-related neurodegenerative diseases. Mitochondrial dysfunction results in Aβ deposition by stimulating mtDNA mutations and ROS production in AD. It can also aggravate the functions and mutations of Parkin, causing PD development. Besides, mHTT impairs mitochondrial ATP production and mitochondrial respiration in HD. SOD1 dysfunction is a cause of sporadic ALS. Secondly, oxidative stress is a key player in aging-related neurodegenerative diseases. Aβ-induced oxidative imbalance and P-tau protein are critical in the neurodegeneration of AD with oxidative stress. Enhanced oxidative stress causes UPS dysfunction and further aggravates the injury of dopaminergic neurons in PD substantia nigra. ROS plays a role in blocking neurotransmitter transmission in HD through stimulating protein misfolding and forming inclusion bodies. Besides, ROS/RNS overproduction is evident in ALS patients. Thirdly, neuroinflammation is implicated in the pathologic processes of aging-related neurodegenerative diseases. The inflammation of AD is mainly regulated by microglia in the innate immune response of the central nervous system. The accumulation and aggregation of α-synuclein in Lewy bodies are involved in the development of PD. The release of inflammatory cytokines induced by mHTT and the activation of NF-kB signaling pathway are the main inflammatory mechanisms of HD. The increased microgliosis and astrocytosis result in decreased MHCI level and ultimately contribute to neurotoxicity in ALS.

With aging, the central nervous system (CNS) gradually undergoes degeneration, which is featured by a chronic and temporary loss of the function and structure of neuronal substances, eventually leading to mental and functional impairment (Campbell et al., 1999). Increasing evidence demonstrates that mitochondrial dysfunction, oxidative stress, and inflammation are primarily pathophysiological mechanisms of aging-related neurodegenerative diseases (Figure 1).

### Mitochondrial Dysfunction

Mitochondria are pivotal modulators of cellular lifecycle, exerting an important role in aging-related neurodegenerative disorders. Emerging evidence suggests that mitochondrial dysfunction shows causal effects in the pathogenesis of these diseases.

#### Mitochondrial Dysfunction and Aging

Mitochondrial dysfunction accelerates aging primarily through two mechanisms, including stimulation of mitochondrial DNA (mtDNA) mutations and generation of reactive oxygen species (ROS). mtDNA mutations, such as point mutations or massive deletions, accumulate with age (Corral-Debrinski et al., 1992). The polymerase chain reaction (PCR) strategy revealed an average of two point mutations per 10 kb in the two protein-coding regions of mtDNA in the elderly subjects, compared with one point mutation per 10 kb in the young brains (Lin et al., 2002). The aggregation of mtDNA mutations with aging is relevant to the mitochondrial dysfunction. In addition, net production of ROS is another momentous mechanism and is considered to be important for mitochondria aging (Munro and Pamenter, 2019).

#### Mitochondrial Dysfunction and Alzheimer’s Disease

Studies have found that the typical histopathological changes of AD are amyloid deposition and neurofibrillary tangles (NFTs). There are many theories trying to explain this change, including the amyloid-β peptide (Aβ) waterfall theory, hyperphosphorylated tau (P-tau) protein theory, neurovascular hypothesis and so on (Ballard et al., 2011). Emerging evidence suggests that mitochondrial dysfunction is associated with AD pathogenesis (Lin and Beal, 2006). In transgenic amyloid precursor protein (APP) mice, oxidative damage preceded Aβ deposition and was associated with upregulation of apoptosis and mitochondrial metabolism related genes (Reddy et al., 2004).

#### Mitochondrial Dysfunction and Parkinson’s Disease

The level of dopaminergic (DA) neurons in the substantia nigra striatum decreases with age. Lewy bodies are unique cytoplasmic inclusion, immunostaining for α-synuclein and ubiquitin. Several causative genes, including DJ-1, PTEN-induced kinase 1 (PINK1), and Parkin (PARK2), strongly support mitochondria dysfunction as a crucial pathogenesis in PD (Lin and Beal, 2006). For example, Chung et al. (2004) found that the functions and mutations of Parkin could be exacerbated by mitochondrial dysfunction.

#### Mitochondrial Dysfunction and Huntington’s Disease

Huntington’s disease is caused by the amplification of CAG triplet repeat in the first exon of huntingtin (HTT) gene, resulting in abnormal forms of the protein to clump together and form aggregates in brain cells. Mitochondrial dysfunction is involved in HD process. Mitochondrial respiration and adenosine triphosphate (ATP) production of striatal cells were obviously impaired in mutant HTT (mHTT) embryos (Milakovic and Johnson, 2005). mHTT can directly act on mitochondria or indirectly affect mitochondrial function via altering transcription (Panov et al., 2002; Sadri-Vakili and Cha, 2006).

#### Mitochondrial Dysfunction and Amyotrophic Lateral Sclerosis

There are 2 types of ALS, among them about 90% are sporadic ALS (SALS) and 10% are familial ALS (FALS). Mutations in superoxide dismutase 1 (SOD1) gene cause 15% of FALS, while SOD1 dysfunction may also be a causal factor of sporadic ALS (Abati et al., 2020). Pathological biopsy of nerves and muscles revealed abnormal mitochondrial structure and localization in ALS. SOD1 mutations cause toxic aggregates formation in mitochondria, thereby disrupting several cellular processes and causing diverse detrimental effects (Hayashi et al., 2016). Animal studies showed that SOD1 mutation overexpression resulted in impaired mitochondrial energy metabolism (Mattiazzi et al., 2002).

### Oxidative Stress

Oxidative stress is an imbalance between biological oxidative and antioxidative systems, which is caused by excessive ROS production (Newsholme et al., 2016). It plays deleterious effects in modulating the function of biomolecules that are sensitive to ROS/reactive nitrogen species (RNS), thus involving neuronal deterioration (Islam, 2017). In addition, the function of heavy metals as antioxidants in oxidative stress and their harmful effects on CNS are indisputable. Oxidative stress is associated with the occurrence and development of aging-related neurodegenerative diseases.

#### Oxidative Stress and Aging

Emerging evidence demonstrates that the free radical theory of aging has gradually become a major mechanism. Oxidative stress cause telomere dysfunction and shortening, which ultimately leads to cell senescence. ROS generation and response to oxidative stress are crucial factors in determining longevity (Finkel and Holbrook, 2000). Antioxidants, such as mental iron, copper, and zinc, are bioaccumulated by various activities. Decreased antioxidants and elevated ROS levels contribute to cellular senescence and thereby lead to various aging-related neurodegenerative diseases.

#### Oxidative Stress and Alzheimer’s Disease

The significance of oxidative stress and ROS in AD through harmful effects on biomolecules has been indicated. It has been reported that accumulation of Aβ aggregates exerts a crucial role in oxidative stress, causing energy failure and mitochondrial dysfunction (Tönnies and Trushina, 2017). Moreover, Aβ-induced oxidative imbalance is associated with increased levels of DNA/RNA oxidation, lipid peroxidation, and protein oxidation, suggesting a central role in AD (Wang X. et al., 2014; Butterfield and Boyd-Kimball, 2018). In addition, emerging evidence proves that metals accumulate in brain with aging and are involved in the pathogenesis of AD. Moreover, P-tau protein, the main constituent of NFTs, is involved in the cognitive decline and neurodegeneration with oxidative stress in AD (Goedert and Spillantini, 2006).

#### Oxidative Stress and Parkinson’s Disease

Motor dysfunction in PD is caused by dopamine depletion in the substantia nigra striatum pathway and dopaminergic neurons loss in the substantia nigra pars compacta (SNpc) (Ramalingam et al., 2019). Oxidative stress exhibits an inevitable effect in progressive neurodegenerative PD (Trist et al., 2019). Dopaminergic neurons are sensitive to mitochondrial ROS (Chen et al., 2019). ROS production is regulated by dopamine metabolism and glutathione in the SNpc (Smeyne and Smeyne, 2013). Patients with PD have enhanced levels of oxidized molecules and reduced glutathione (Homma and Fujii, 2015). The ubiquitin-proteasome system (UPS) reduces oxidative free radicals production (Collier et al., 2011). Enhanced oxidative stress lead to UPS dysfunction and further aggravate the injury and damage of dopaminergic neurons in PD substantia nigra (Betarbet et al., 2005).

#### Oxidative Stress and Huntington’s Disease

Although the primary cause of HD has been proved to be the toxicity of mHTT, diverse other processes have also been shown to be associated with HD (e.g., oxidative stress). Singh et al. (2019) revealed that oxidative stress exerts a vital role in neuronal degeneration cascade in HD. ROS causes the formation of inclusion bodies by inducing protein misfolding, which clump together in neurons and block neurotransmitter delivery (Rubinsztein and Carmichael, 2003). Repair of damaged DNA may result in instability and amplification of CAG nucleotide repeats in mHTT (Kolli et al., 2017).

#### Oxidative Stress and Amyotrophic Lateral Sclerosis

Studies have shown that oxidative stress regulated a range of cellular biological and pathological processes, including lipid peroxidation, protein injury, as well as DNA and RNA oxidation in ALS (Morimoto et al., 2020). SOD1 mutations and ROS/RNS overproduction are evident in ALS (Baltazar et al., 2014). Besides, the oxidative stress biomarkers can bind to biomolecules, such as malondialdehyde (MDA) modified protein and lipid peroxidation product in ALS (Mendez and Sattler, 2015).

### Inflammation

Neurodegeneration, featured by the loss and progressive dysfunction of axons and neurons, is the main pathological feature of neurodegenerative disorders (Amor et al., 2010). Recent studies have shown the function of neuroinflammation in neurodegeneration, especially in the elderly. There is a growing awareness that inflammation is involved in the pathological process of aging-related neurodegenerative diseases. Although triggering events are varied, chronic immune activation is a common feature. Immune activation in the CNS exerts a pivotal role in the modulation of brain homeostasis during development and aging. When responding to tissue damage and pathogen invasion, nerve cells continuously survey the microenvironment and promote the inflammatory response, thereby further engaging self-limiting reaction through the immune system and starting tissue repair (Wyss-Coray and Mucke, 2002).

#### Inflammation and Aging

Inflammation plays a double-edged sword in the process of aging. Advantageously, it is closely associated with immunity by resisting pathogen invasion. Detrimentally, an excessive inflammatory response can disrupt the balance of the organism, which may eventually lead to disease. “Neuroinflammatory aging” is related with an obvious reduction of neuron numbers, neuronal dendrites, cortex and spine volume. The brain barrier is a structure that maintain the normal functional activities of neurons in the CNS, which is composed of cerebrospinal fluid-brain barrier (CBB), blood–brain barrier (BBB), and blood-cerebrospinal fluid barrier (BCB) (Abbott et al., 2010). The neuroinflammation sensitivity of different barriers and brain regions is significantly different (Stephenson et al., 2018). Changes in cells activation during aging explain the incremental susceptibility of the elderly to neuroinflammation and neurodegenerative diseases (Benz and Liebner, 2020).

#### Inflammation and Alzheimer’s Disease

Considerable evidence suggests that systemic inflammation is closely related to the pathogenesis of AD (Holmes, 2013). Systemic inflammation in AD could increase the expression of central proinflammatory cytokines, the death of neuronal cells, and the generation of ROS (Perry et al., 2010), thereby exacerbating the clinical symptoms. Moreover, the permeability of BBB gradually increases the progression of AD (Farrall and Wardlaw, 2009). The harmful effects of peripheral inflammation are associated with central inflammatory response because of the crosstalk between the periphery and the CNS. Therefore, it suggests that peripheral inflammation is a potential risk factor for AD progression. Inflammation in AD is primarily regulated by microglia in the innate immune responses of the CNS.

#### Inflammation and Parkinson’s Disease

Since PD-like symptoms were first observed in individuals infected with influenza virus, the function of inflammation in PD has been widely studied (Lutters et al., 2018). Inflammation contributes to the occurrence and development of PD both directly or indirectly. Studies have found that some viral proteins could promote the accumulation and aggregation of α-synuclein in Lewy bodies (Maries et al., 2003), further suggesting brain inflammation in the pathogenesis of PD. In addition, neurotropic pathogens could reach the basal ganglia via diverse pathways, and ultimately cause a series of neuroinflammation and neurodegenerative disorders in the nigrostriatal tract (Hawkes et al., 2007).

#### Inflammation and Huntington’s Disease

There is a growing interest of inflammation in HD progression. It has been demonstrated the higher expression of mHTT in monocytes and microglia (Moscovitch-Lopatin et al., 2010). Increasing evidence proves that mHTT influences the function of monocytes and microglia by mediating inflammation in HD (Valadão et al., 2020). On the one hand, mHTT directly induces the release of inflammatory cytokines (Gupta et al., 2021). On the other hand, mHTT promotes the release of inflammatory chemokines or cytokines by modulating nuclear factor-κB (NF-κB) signaling pathway (Khoshnan et al., 2004). Neuronal damage itself can cause a vicious cycle of inflammatory response and neurodegenerative events, which in turn leads to more neuronal death in HD (Zuccato et al., 2010). Moreover, a pioneer study observed the high levels of interleukin-6 (IL-6), IL-8, IL-10 and tumor necrosis factor-α (TNF-α) in HD patients, all of which were associated with disease progression (Silvestroni et al., 2009).

#### Inflammation and Amyotrophic Lateral Sclerosis

Neuroinflammation is a key modulator of ALS progression, and is featured by astroglia, microglia, infiltrating lymphocytes and peripheral monocytes in CNS (Lyon et al., 2019). Emerging evidence shows that inflammation of the innate immune system is linked to ALS (Lu et al., 2016). Major histocompatibility complex class I (MHCI) expression is increased in neuromuscular junction (NMJ) and peripheral motor axons and NMJs in SOD1 mutation mice (Nardo et al., 2016). Increased MHCI level exhibits a neuroprotective effect in peripheral nerves, depending on the removal of motor axon debris by immune cells (Thonhoff et al., 2018). MHCI level is decreased because of the increased microgliosis and astrocytosis in SOD1 mutation mice and ALS patients, ultimately promoting neurotoxicity (Song et al., 2016).

## LncRNAs and Aging

Aging is a natural phenomenon featured by accumulation of degenerative alterations and damage. It is a primary risk factor in the etiology and development of various diseases, such as metabolic disruptions, neurodegenerative disorders, cardiovascular malfunctions, and cancers. Progressive loss of the functions of multiple cells, tissues, or organs is positively associated with aging. Though diverse mechanisms of aging have been studied, such as telomere shortening, free radicals, accumulated mutations, defective DNA repair, and increased DNA damage, the aging process remains widely unknown (Sinha et al., 2014). In fact, the current knowledge underscores the significance of multiple theories of aging. Therefore, it is best defined as a multifactorial process that involves complex interacting molecular and cellular mechanisms. Accordingly, there is currently no single measure that can be qualified as a specific biomarker or hallmarks of aging. A growing number of studies are exploring multiple biomarkers of aging at different levels, providing potential prospects for clinical diagnosis and therapy.

Cellular senescence is a causative process of aging and is responsible for aging-related diseases. It is a permanent state of cell cycle arrest, accompanied by an enhanced secretory phenotype and resistance to cell death. Cellular senescence can be induced by telomere attrition, DNA damage, mitochondrial dysfunction, chromosome destabilization, and oncogene activation (Wei and Ji, 2018). There are no specific or universal hallmarks for senescent cells. Several senescence biomarkers can be used to assess cellular senescence, such as higher activities of senescence-associated β-galactosidase (SA-β-gal), changes in senescence-related proteins (p16, p21, p27, and p53), alterations in cellular senescence-related morphology, and production of senescence-associated secretory phenotype (SASP) (Calcinotto et al., 2019; Ni et al., 2020). There is growing interest in discovering novel markers of senescence.

LncRNAs, a class of ncRNAs ≥ 200 nt in length, play an important role in many biological processes, such as transcription, post-transcriptional processing, and chromatin modification. According to their genomic location and orientation, they can be divided into sense, antisense, bidirectional, intergenic, intron, enhancer, and promoter lncRNAs (He et al., 2018; Cui et al., 2021). Mammalian transcription generates various lncRNAs involved in organ development (Rayner and Liu, 2016), differentiation (Chen and Zhang, 2016), synaptic formation (Maag et al., 2015), learning and memory (Gudenas and Wang, 2015). Increasing evidence has shown that a large number of lncRNAs are involved in cellular senescence at diverse stages of the cell cycle (Puvvula, 2019). With the accumulated evidence, we hereby discuss the association and correlation between lncRNAs and aging.

Firstly, lncRNAs play a role in cellular senescence and organismal aging by regulating cell cycle. For example, studies have demonstrated that depletion of metastasis-associated lung adenocarcinoma transcript 1 (MALAT1) could induce G1 or G1/S arrest, thereby enhancing senescence phenotype and inhibiting cell growth (Tripathi et al., 2013). LncRNA H19 is necessary in cell proliferation, growth, and senescence. Ratajczak (2012) proved that imprinting deletion of insulin-like growth factor-2 (Igf2)-H19 locus was involved in cellular senescence. Besides, p21-associated ncRNA DNA damage activated (PANDA) has been reported to trigger DNA damage via p53, leading to G1 cell cycle arrest (Wang Y. et al., 2017). In addition, several other lncRNAs are associated with aging process by affecting cell cycle, including Gadd7, 7SL, FAL1, etc. (Liu et al., 2012; Kour and Rath, 2016).

Secondly, lncRNAs participate in the progression of SASP and promote the secretion of inflammatory factor. LncRNA nuclear paraspeckle assembly transcript 1 (NEAT1) is regarded as a novel inflammatory modulator. It can influence the formation of paraspeckles and subsequently regulate cellular senescence (McCluggage and Fox, 2021). Moreover, lncRNA LET stimulates the accumulation of nuclear factor 90 (NF90), which inhibits the translation of several SASP factors (Tominaga-Yamanaka et al., 2012; Yang et al., 2013). Cui et al. (2014) revealed that lnc-IL7R could alleviate the inflammatory response induced by lipopolysaccharide, suggesting an involvement in SASP production. Additionally, lincRNA-Cox2, Lethe, and THRIL have been shown to contribute to the generation of SASP (Panda et al., 2017).

Thirdly, lncRNAs is involved in telomere dynamics and attrition. TElomere Repeat-containing RNA (TERRA) is a lncRNA transcribed by telomeric DNA sequences. Many studies have been conducted on how TERRA transcription modulates telomere structure (Aguado et al., 2020). Yehezkel et al. (2013) confirmed an association between TERRA and aging process. They found that TERRA expression was upregulated, telomere shortening was accelerated, and replicative senescence enters prematurely in immunodeficiency, centromeric instability and facial anomalies (ICF) syndrome type I. Moreover, telomeric RNA component (TERC), a 451 nt lncRNA, is the core component of telomerase with classical function to provide template for the extension of telomerase. Introduction of TERC rescued premature aging phenotype of telomerase deficient mice, suggesting a role of TERC in senescence and aging (Samper et al., 2001).

Lastly, lncRNAs contribute to recruitment of chromatin remodeling complexes during senescence and aging. HOX antisense intergenic RNA (HOTAIR) plays a role in the recruitment and binding of chromatin remodeling complexes to the HOX sites, leading to retargeting of polycomb repressive complex 2 (PRC2) (Rinn et al., 2007). Besides, KCNQ-overlapping transcript 1 (Kcnq1ot1) has been reported to affect Kcnq1 locus by interacting with PRC2 complexes (Pandey et al., 2008). Additionally, recent studies have demonstrated that several other lncRNAs, such as Air, H19, and TERRA are also involved in chromatin remodeling (Zhu et al., 2013; He et al., 2018).

## Roles of lncRNAs in Aging-Related Neurodegenerative Diseases

Aging-related neurodegenerative diseases are a range of progressive atrophy and loss of neurological function of neurons and neural tissues, eventually leading to cognitive or motor impairments. Although common mechanisms may result in neuron loss in a variety of disorders, different pathological characteristics are caused by specific toxic aggregation of particular proteins and/or genetic mutations. For example, Aβ aggregation and P-tau in AD, α-synuclein Lewy bodies in PD, mHTT aggregates in HD and SOD1 in ALS (Chung et al., 2011). Increasing evidence indicates that diverse lncRNAs are involved in neural function, and their related RNA networks may influence neurodegeneration (Wu and Kuo, 2020). Since the pathology of neurodegenerative diseases are related to accumulation of certain proteins, studies have suggested that lncRNAs are associated with different protein aggregation events and disease pathogenesis. Abnormal regulation of lncRNAs is related to the aging process and aging-related diseases. In the following sections, we summarize the roles of lncRNAs in aging-related AD, PD, HD, and ALS.

### Roles of lncRNAs in Alzheimer’s Disease

**FIGURE 2:**
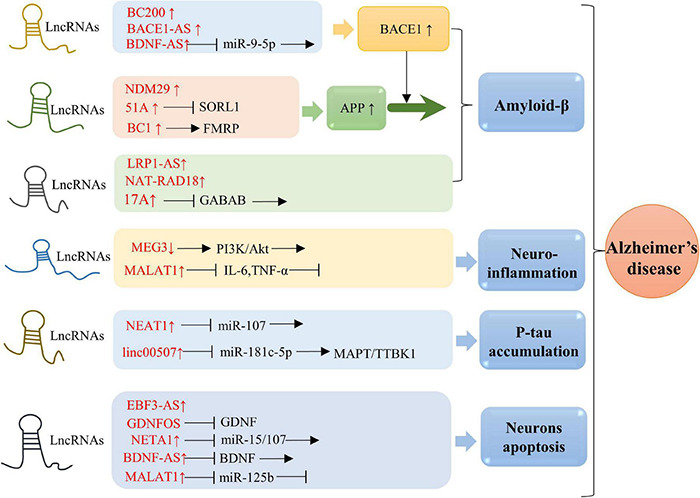
Molecular mechanisms of lncRNAs in AD. Overexpression of BACE1-AS, BC200, and BDNF-AS is associated with BACE1 activity. Elevated level of 51A in AD patient altered splicing mode of SORL1, causing damage to APP processing and leading to promotion of the Aβ deposition. Upregulation of BC1 induces APP mRNA translation by binding to FMRP. Enhanced expression of NDM29 induces APP synthesis. Overexpression of LRP1-AS is involved in regulating Aβ accumulation. NAT-RAD18 level is upregulated in response to Aβ40. Overexpression of 17A is tightly associated with Aβ secretion and Aβ42 production. Down-expression of MEG3 promotes neuroinflammation via PI3/Akt pathway. Significantly upregulated expression of MALAT1 inhibits neuroinflammation through reducing IL-6 and TNF-α. NEAT1 and linc00507 have been characterized to be involved in Tau. Upregulation of NEAT1 promoted Tau protein phosphorylation by sponging miR-107, while elevated level of linc00507 enhances hyperphosphorylation of Tau protein through regulating miR-181c-5p/MAPT/TTBK1 pathway. Upregulation of NETA1 and MALAT1 promoted cell apoptosis by targeting miR-15/107 and miR-125b, respectively. In addition, EBF3-AS exhibits a role in regulating neurons apoptosis.

Alzheimer’s disease, the major etiology of dementia, is characterized by aggregation of Aβ peptides and P-tau in neurofibrillary tangles. APP is a transmembrane protein that produces Aβ by sequential division of β-site APP cleaving enzyme-1 (BACE1) and γ-secretase (Karran et al., 2011). The balanced ratio of Aβ42/Aβ40 is disrupted and cause the amyloid plaques in AD brain (Ballard et al., 2011). It has been demonstrated that multiple lncRNAs are involved in AD pathology (Figure 2). In this section, we compile the roles of lncRNAs in aging-related AD (Table 1).

**TABLE 1:** Roles of lncRNAs in ageing-related Alzheimer’s disease.

Disease	LncRNAs	Expression	Functions	References
AD	BACE1-AS	↑	Increase BACE1 mRNA stability, generate Aβ42, interact with HuD.	Deschênes-Furry et al., 2006; Faghihi et al., 2008
	BC200	↑	Promote BACE1 activity and expression, induce plasticity failure.	Mus et al., 2007; Feng et al., 2018; Li et al., 2018
	BC1	↑	Induce Aβ peptide accumulation, cause memory and spatial learning impairments.	Zhang et al., 2018
	NEAT1	↑	Enhance Aβ and P-tau level, induce neuronal death.	Ke et al., 2019
	MALAT1 (NEAT2)	↑	Inhibit neuron apoptosis and neuroinflammation.	Ma et al., 2019
	17A	↑	Enhance the Aβ42/Aβ40 peptide ratio, deactivate GABAB signaling.	Massone et al., 2011
	51A	↑	Interact with APP, promote Aβ formation.	Ma et al., 2009; Ciarlo et al., 2013
	BDNF-AS	↑	Enhance apoptosis and decrease cell viability, act as ceRNA to promote neurotoxicity.	Guo et al., 2018; Ding et al., 2021
	SOX2OT	↑	Reduce neurogenesis.	Arisi et al., 2011
	GDNFOS	Dysregulated	Regulate endogenous GDNF level.	Airavaara et al., 2011
	EBF3-AS	↑	Regulate neuron apoptosis.	Gu et al., 2018
	NDM29	↑	Induce APP synthesis, promote cleavage.	Massone et al., 2012
	LRP1-AS	↑	Regulate Aβ accumulation.	Yamanaka et al., 2015
	linc00507	↑	Promote p-Tau accumulation.	Yan et al., 2020
	MEG3	↓	Reduce Aβ expression, decrease inflammation.	Yi et al., 2019
	NAT-RAD18	↑	Induce defective DNA repair.	Parenti et al., 2007

BACE1 has been extensively recognized as the potential to develop Aβ-lowering drug therapy for AD. BACE1 antisense transcript (BACE1-AS) is a conserved lncRNA transcribed from the antisense protein-coding BACE1 gene (Fotuhi et al., 2019). Faghihi et al. (2008) reported that BACE1-AS realizes its function via raising BACE1 mRNA stability and then producing additional Aβ42, suggesting the driving function of BACE1-AS in AD pathologic process. They also demonstrated that BACE1-AS could prevent binding to miR-485-5p. HuD, one of the main neuronal RNA-binding proteins (RBPs), is involved in mediating neuronal maintenance, differentiation and plasticity, thus regulating memory and learning. HuD could interact with BACE1 mRNA, APP mRNA, and BACE1-AS to prolong the half-lives of the mRNA and enhance the expression of BACE1 (Deschênes-Furry et al., 2006). Kang et al. (2014) found elevated expressions of HuD and BACE1-AS in AD brains.

Brain cytoplasmic 200 (BC200) is a major cytoplasmic lncRNA, which is mainly expressed in neurons and can be transported to dendrites. It is a translational modulator that selectively targets the somatic dendrite domain of neurons. BC200 is believed to act as a regulator of local protein synthesis in the postsynaptic dendrite microdomain and is involved in maintaining long-term synaptic plasticity (Mus et al., 2007). BC200 has been reported to exhibit important effects in human lung, esophagus, and cervix tumors (Hu and Lu, 2015). Mus et al. (2007) proved that BC200 RNA expression was markedly specific increased in AD brains. They also indicated that elevated BC200 levels was paralleled with the severity of AD. In addition, studies have shown that BC200 upregulation directly promote BACE1 level and impair cell viability subsequently, thereby increasing Aβ42 expression (Feng et al., 2018; Li et al., 2018).

Brain cytoplasmic 1 (BC1) contains a 5′ stem-loop domain, followed by a single-stranded central homopolymer A-rich region and a 3′-stem-loop domain (Lin et al., 2008). BC1 acts as a translational repressor, which is modulated by the adjacent A-rich region and 3′ stem-loop through interactions with poly(A)-binding protein (PABP), eukaryotic initiation factor 4A (eIF4A), and eIF4B (Kondrashov et al., 2005; Lee et al., 2020). It is abundant in the synapse and inhibits translation at initiation. BC1 is a cytoplasmic lncRNA in neurons that promotes APP mRNA translation through the interaction with the fragile X syndrome protein (FMRP) (Aleshkina et al., 2021). Zhang et al. (2018) demonstrated that inhibition of BC1 blocked the accumulation and aggregation of Aβ in AD mice brains and protected them from memory and learning deficits. In contrast, exogenous overexpression of BC1 caused Aβ peptides aggregation and induced learning and memory disorders.

NEAT1 consists of two subtype transcripts, NEAT1v1 and NEAT1v2, which are essential components of para-nuclear plaque formation (Koyama et al., 2020). LncRNA NEAT1 has been reported to be widely expressed in many mammalian cells. Increasing evidence demonstrates that NEAT1 plays crucial roles in multiple pathophysiological processes including neurodegenerative diseases (An et al., 2018), cancers (Yu et al., 2017), immune disorders (Taheri et al., 2020), and *etc*. Previous studies have shown that NEAT1 is involved in the occurrence of AD. Ke et al. (2019) found that NEAT1 exacerbated P-tau expression, Aβ level, and neuron damage via sponging miR-107, thus boosting AD progression. Besides, it has been reported that NEAT1 is involved in AD through modulating the miR-124/BACE1 signaling pathway (Zhao et al., 2019). Additionally, Spreafico et al. (2018) indicated that NEAT1 facilitated neuronal cell death via regulating the expression of microRNA-15/107 family in postmortem AD tissues. MALAT1, also known as NEAT2, has recently been implicated in neurodegenerative diseases due to anti-inflammatory property (Masoumi et al., 2019). Ma et al. (2019) indicated that MALAT1 interacted with miR-125b to promote neurite growth while inhibit neuronal apoptosis and suppress inflammatory cytokines in AD.

LncRNA 17A is synthesized by RNA polymerase III and located at intron 3 of g protein-coupled receptor 51 gene (Luo and Chen, 2016). LncRNA 17A could damage gamma-aminobutyric acid type B (GABAB) signal transduction by generating non-functional receptor isoforms, enhance the Aβ42/Aβ40 peptide ratio, and promote neurodegeneration (Massone et al., 2011). Moreover, lncRNA 17A expression has been found to be upregulated in AD patients. Besides, inflammation of brain tissue triggers 17A expression and complicates the AD process (Wang X. et al., 2019).

LncRNA 51A locates in an antisense configuration on intron 1 of the neuronal sortilin-related receptor (SORL1) gene. SORL1 has been reported to interact with APP, affect transport and proteolysis, and participate in AD pathogenesis (Ciarlo et al., 2013). Decreased SORL1 level shifts APP from the reverse transcriptional cycle to the β-secretase cleavage pathway, thus increasing APP secretion and subsequent Aβ formation. It has been proposed that overexpression of 51A promotes Aβ formation by decreasing SORL1 variant A, thereby increasing susceptibility to AD (Ma et al., 2009).

Brain-derived neurotrophic factor (BDNF), a member of the neurotrophic factor family, is a widely studied growth factor which includes neurotrophin-3 (NT3), neurotrophin-4 (NT4), and nerve growth factor (NGF) (Björkholm and Monteggia, 2016). BDNF has a profound impact on brain morphology, development, and function because it is widely expressed in the CNS. BDNF-antisense (BDNF-AS) is a lncRNA with dozens of alternate splicing variants transcribed from 11p14 cytogenetic band. LncRNA BDNF-AS continuously reduces endogenous BDNF protein levels and functions by altering the chromatin structure of the BDNF region, thereby inhibiting the expression of BDNF sense transcripts (Modarresi et al., 2012). It is involved in a variety of processes in the CNS, including synaptic plasticity, synapse formation, and neuronal maturation. Abnormal regulation of BDNF-AS is implicated in AD process. Guo et al. (2018) found an increased level of BDNF-AS and a reduced expression of BDNF, accompanied by enhanced apoptosis induction and decreased cell viability in an AD cell model established by Aβ_25–35_ exposure to PC12 cells. They also demonstrated that BDNF-AS silencing had crucial beneficial effects on enhancing cell viability, and suppressing oxidative stress and apoptosis through negative modulation of BDNF. Ding et al. (2021) uncovered an elevated expression of BDNF-AS in the peripheral blood of AD patients. Further mechanism studies showed that BDNF-AS acted as a ceRNA to competitively bind miR-9-5p to induce BACE1 expression, ultimately promoting neurotoxicity.

The human SOX2 gene encodes a protein of 317 amino acids (Stevanovic et al., 1994). The structural core of SOX2 is its high-mobility-group (HMG) domain, which contains a nuclear export signal and a nuclear localization in addition to binding to specific DNA consensus sequences (Novak et al., 2020). The transcription factor SOX2 is a single exon protein, which exerts an essential and pleiotropic role in development and homeostasis. LncRNA SOX2 overlapping transcript (SOX2OT) is located at 3q26.3 on human chromosome and is constitutive of more than two transcription start sites and ten exons (Wang et al., 2020). The intronic region of SOX2OT contains a SOX2 structure and shares the same transcriptional direction (Fantes et al., 2003). Arisi et al. (2011) revealed that SOX2OT reduces neurogenesis by mediating SOX2 gene expression in AD. It has been proved that abnormal expression or activation of 10–11 translocation-2 (TET2) is closely associated with AD (Carrillo-Jimenez et al., 2019). Li L. et al. (2021) demonstrated that TET2 was involved in neuron formation via modulating several lncRNAs (SOX2OT, MALAT1, *etc*.).

Glial cell line-derived neurotrophic factor (GDNF) is a 134 amino acid protein in the GDNF family ligands (GFLs) including artemin, persephin, and neurturin (Cintrón-Colón et al., 2020). It is an effective nutrient factor for central norepinephrine neurons, midbrain dopaminergic neurons, spinal motoneurons and peripheral neurons. GDNF opposite strand (GDNFOS), as its name suggests, is transcribed from the opposite strand of GDNF gene in the primate genome. GDNFOS isoforms have been found to be differentially expressed in tissue expression patterns and could regulate the level of endogenous GDNF in AD brains (Airavaara et al., 2011).

Early B cell factor 3 (EBF3) belongs to the Collier/Olf/EBF (COE) transcription factor family. It is an evolutionarily conserved atypical transcription factor thought to affect the stratification of cerebral cortex. Gu et al. (2018) revealed that lncRNA EBF3 antisense (EBF3-AS) expression was enhanced in hippocampus of AD mice. Moreover, they also found that EBF3-AS was involved in the regulation of neurons apoptosis in AD cell models induced by okadaic acid (OA) and Aβ_25–35_.

Neuroblastoma differentiation marker 29 (NDM29) is a lncRNA transcribed by RNA polymerase III. NDM29 markedly enhances APP level, which in turn increases generation of the two major Aβ isoforms (Li D. et al., 2021). LncRNA NDM29 was reported to induce APP synthesis and accelerate the cleavage through γ-secretase and BACE1, which can be restrained by anti-inflammatory agents and accelerated by inflammatory stimulation (Massone et al., 2012).

Low-density lipoprotein receptor (LDLR)-related protein (LRP1) is a large endophagocytic and signal transduction receptor in the LDLR gene family. It is widely expressed in the brain. Apolipoprotein E (ApoE), the ligand of LRP1, is involved in senile plaques in AD brains (Rebeck et al., 1993), implicating a role for LRP1 in the accumulation of Aβ. LRP1-antisense (LRP1-AS) negatively mediates LRP1 expression at both protein and RNA levels. It was reported that LRP1-AS expression was significantly increased in the AD brain (Yamanaka et al., 2015).

In addition, a variety of other lncRNAs exhibit an indispensable role AD progression. For instance, Yan et al. (2020) demonstrated that elevated linc00507 level in AD models promoted P-tau accumulation through miR-181c-5p/microtubule-associated protein tau (MAPT)/tau-tubulin kinase-1 (TTBK1) network. LncRNA MEG3 is an imprinted gene mapped in human chromosome 14 and mouse chromosome 12. Yi et al. (2019) found a downregulated level of MEG3 in AD mice. They proved that upregulation of MEG3 could decrease Aβ expression, reduce inflammation injury, and protect neurons by phosphatidylinositol 3-kinase (PI3K)/protein kinase B (Akt) pathway. RAD18 is a member of the chromosome family responsible for repairing DNA damage (Hedglin and Benkovic, 2015). NAT-RAD18 is a lncRNA of the natural antisense transcript against RAD18. It was found that RAD18 expression was down-regulated, while NAT-RAD18 level was up-regulated in response to Aβ40 (Parenti et al., 2007).

### Roles of lncRNAs in Parkinson’s Disease

**FIGURE 3:**
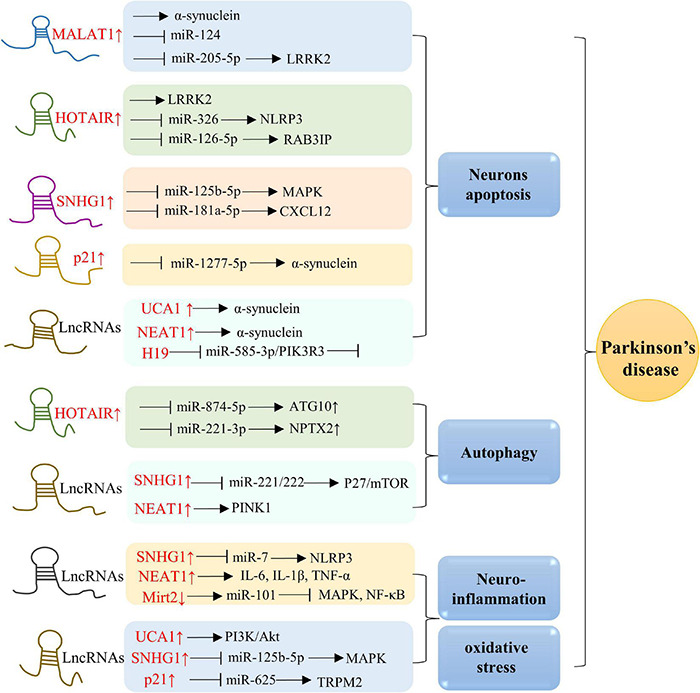
Molecular mechanisms of lncRNAs in PD. Overexpression of MALAT1, HOTAIR, SNHG1, p21, UCA1, NEAT1, and H19 are in associations with neurons apoptosis. Besides, lncRNAs, including HOTAIR, SNHG1, and NEAT1, also play a role in modulating autophagy. Additionally, elevated levels of SNHG1, NEAT1, Mirt2, UCA1, and p21 in AD are positively related to oxidative stress and neuroinflammation.

Parkinson’s disease is pathologically characterized by α-synuclein aggregation in Lewy bodies. Several site mutations are recognized to cause hereditary PD, including α-synuclein, Parkin, DJ-1, LRRK2, PINK1, and ATP13A2 (Majidinia et al., 2016). Neurotoxins such as 6-hydroxydopamine (6-OHDA) and MPTP/MPP+ have been often utilized to induce degeneration dopaminergic neuron degeneration and reproduce pathological features of PD (Nagel et al., 2009). Accumulating evidence proposes that lncRNAs are closely related to the development of PD (Figure 3). In this section, we discuss the roles of lncRNAs in aging-related PD (Table 2).

**TABLE 2:** Roles of lncRNAs in aging-related Parkinson’s disease.

Disease	LncRNAs	Expression	Functions	References
PD	MALAT1	↑	Promote α-synuclein proteostasis, neuroinflammation, autophagy and neuroapoptosis.	Zhang et al., 2016; Liu et al., 2017; Chen et al., 2018
	HOTAIR	↑	Enhance LRRK2 level, induce neuronal injury, apoptosis, autophagy.	Wang S. et al., 2017; Lin et al., 2019; Lang et al., 2020; Zhao et al., 2020; Zhang et al., 2021
	SNHG1	↑	Promote neuronal damage, increase cell death, regulate mTOR phosphorylation, promote neuroinflammation and oxidative stress.	Cao et al., 2018; Qian et al., 2019; Wang et al., 2021; Xiao et al., 2021
	UCA1	↑	Promote α-synuclein expression, induce neuronal damage, oxidative stress and inflammation.	Lu M. et al., 2018; Cai et al., 2019
	p21	↑	Mediate α-synuclein, neuroinflammation, mitochondrial dysfunction and oxidative stress.	Xu et al., 2018; Ding et al., 2019
	NEAT1	↑	Promote α-synuclein associated apoptosis, promote neuroinflammation.	Liu and Lu, 2018; Yan et al., 2018
	Mirt2	↓	Block MAPK and NF-κB cascades.	Han et al., 2019
	H19	↑	Mediate neuronal apoptosis.	Zhang et al., 2020
	MAPT-AS1	↓	Regulate MAPT expression.	Coupland et al., 2016

The original sequence of MALAT1 gene is over 8,000 bp, which is highly conserved in 33 species of mammals (Ji et al., 2003). It is also named as NEAT2. Given that MALAT1 is expressed in a variety of nerve cells in the brain, it is not surprising that MALAT1 exerts diverse roles in normal brain physiology. Increasing evidence observes dysregulated MALAT1 in PD progression. MALAT1 is upregulated in MPP+ induced PD cells and MTPT-induced PD mice (Kraus et al., 2017). Studies have found that MALAT1 participates in PD pathology through regulating various mechanisms, including α-synuclein proteostasis, neuroinflammation, autophagy, and neuroapoptosis. LRRK2 mutation is one of the causes for inherited and sporadic PD (Jankovic and Tan, 2020). Liu et al. (2017) found a reversed expression between MALAT1 and miR-124 in MPTP-induced PD mice. Further mechanistic studies showed that MALAT1 promoted apoptosis by interacting with miR-124 and negatively modulating its expression. Zhang et al. (2016) proved that MALAT1 could increase the expression of a-synuclein protein by binding with it to enhance the stability. Besides, Chen et al. (2018) revealed elevated levels of MALAT1 and LRRK2, and decreased expression of miR-205-5p in MPTP-induced PD mice. They proposed that the MALAT1/miR-205-5p axis modulates cellular apoptosis by targeting LRRK2.

LncRNA HOTAIR is transcribed from the antisense chain of homologous frame C locus on chromosome 12. HOTAIR has been extensively explored in human cancer (Rajagopal et al., 2020). Molecular mechanisms of HOTAIR in cancer progression include recruitment of lysine specific demethylase 1 (LSD1) complexes and PRC2, histone 3 lysine 4 (H3K4) demethylation and histone 3 lysine 27 (H3K27) methylation (Qu et al., 2019). Recent studies have found the functions of HOTAIR in PD progression. HOTAIR has been reported to accelerate MPP+-induced neuron damage by mediating the miR-874-5p/autophagy-related 10 (ATG10) axis in PD (Zhao et al., 2020). Besides, Wang S. et al. (2017) demonstrated that HOTAIR could enhance the expression and stability of LRRK2, thus involving in PD process. Rab3a interacting protein (RAB3IP), is a Rab-specific guanine nucleotide exchange factor (GEF) (Tong et al., 2021). Lin et al. (2019) indicated the function of the cellular HOTAIR/miR-126-5p/RAB3IP signaling pathway in PD. NOD-like receptor family pyrin domain containing 3 (NLRP3) is a prominent inflammasome in immune system and contributes to several disease. HOTAIR targeted miR-326 to promote the neuronal injury through facilitating NLRP3 mediated apoptosis in PD (Zhang et al., 2021). Additionally, Lang et al. (2020) explored that HOTAIR enhanced neuronal pentraxin II (NPTX2) through targeting miR-221-3p, thereby driving autophagy in PD mouse models.

LncRNA small nucleolar RNA host gene 1 (SNHG1), also known as linc00057, is approximately 3,927 bases in length (Tycowski et al., 1994). Studies has focused on its roles as a competing endogenous RNA (ceRNA) to modulate tumorigenesis (Lu Q. et al., 2018). SNHG1 attenuates p53 expression, thus promoting cell migration, proliferation, and invasion (Shen et al., 2017). Notably, SNHG1 has been recognized as a key modulator of PD neurotoxicity. The C-X-C motif chemokine ligand 12 (CXCL12) binds to its receptors to induce downstream signaling pathways (Shi et al., 2020). Wang et al. (2021) demonstrated that SNHG1 stimulated MPP+ induced neuronal damage through mediating miR-181a-5p/CXCL12 axis. Besides, Qian et al. (2019) indicated another mechanistic pathway by which SNHG1 targets miR-221/222 and subsequently modulates p27/mammalian target of rapamycin (mTOR) expression in MPP+-induced PD models. Moreover, SNHG1 regulates PD by participating in miR-7/NLRP3 pathway to promote neuroinflammation (Cao et al., 2018). Moreover, Xiao et al. (2021) proved that SNHG1 could also promote oxidative stress, apoptosis, and inflammation by modulating the miR-125b-5p/mitogen-activated protein kinase 1 (MAPK1) axis in models of PD.

LncRNA urothelial carcinoma-associated 1 (UCA1) was first identified in human bladder transitional cell line (Wang et al., 2006). Increasing evidence demonstrates that UCA1 is involved not only in many tumor diseases, but also in regulating neurodegenerative disorders. LncRNA UCA1 has been widely studied in the pathogenesis and development of PD. Studies have demonstrated that UCA1 participates in regulating α-synuclein aggregation, dopaminergic neuroapoptosis, and neuroinflammation. Lu M. et al. (2018) explored that UCA1 promoted the expression of a-synuclein in PD development. Besides, Cai et al. (2019) showed that UCA1 could aggravate dopaminergic neuronal damage, inflammation, and oxidative stress by promoting the PI3K/Akt axis, while reduction of UCA1 exerted opposite effects in a PD rat model.

LncRNA-p21 has been studied to regulate PD via mediating α-synuclein, mitochondrial dysfunction, oxidative stress and neuroinflammation. Xu et al. (2018) indicated that lncRNA-p21 stimulated cellular apoptosis and suppressed cell viability by sponging miR-1277-5p and indirectly enhancing α-synuclein level in PD. The transient receptor melastatin 2 (TRPM2) is a non-selective Ca^2+^ osmotic channel elevated in PD brains (Belrose and Jackson, 2018). It has been found that lncRNA-p21-miR-625-TRPM2 axis has pivotal effects in oxidative stress and neuroinflammation in PD models (Ding et al., 2019).

LncRNA NEAT1 is also referred to PD progression. Overexpression of NEAT1 was positively correlated with the concentration of MPTP and promoted the stability of PINK1 protein (Yan et al., 2018). Mechanistically, NEAT1 positively modulated PINK1 level through stabilizing PINK1 protein and suppressing PINK1 protein degradation. The levels of TNF-α, IL-6, and IL-1β, were up-regulated in MPP+-induced PD models, implying that NEAT1 is closely correlated with neuroinflammation (Yan et al., 2018). In addition, Liu and Lu (2018) showed that NEAT1 could promote α-synuclein-related apoptosis in PD.

In addition, many other lncRNAs play important roles PD process. For example, myocardial infraction associated transcript 2 (Mirt2) exerts anti-inflammatory effects in many cell types, while PD is often associated with excessive inflammation. Therefore, Han et al. (2019) proved that miR-101 inhibited by Mirt2 led to the blocking of MAPK and NF-κB cascades, which might be important in the treatment of PD. Besides, Zhang et al. (2020) demonstrated that lncRNA H19 mitigated neuronal apoptosis by targeting miR-585-3p/phosphoinositide-3-kinase regulatory subunit 3 (PIK3R3) in MPP+ treated neuroblastoma cells and MPTP-induced PD mice. LncRNA microtubule-associated protein tau antisense RNA 1 (MAPT-AS1) locates on the antisense chain of the promoter region of MAPT, which is believed to associated with disease state of PD (Wang D. et al., 2019). It has been found that MAPT-AS1 is an underlying epigenetic modulator of MAPT expression in PD (Coupland et al., 2016).

### Roles of lncRNAs in Huntington’s Disease

Huntington’s disease is a neurodegeneration caused by a CAG repeat in the gene encoding HTT protein (Jimenez-Sanchez et al., 2017). Brain-derived neurotrophic factor (BDNF), a neurotransmitter regulator, is involved in neuroplasticity and is critical for its survival and growth (Sharma et al., 2021). It has been found that BDNF expression reduces in HD (Humbert and Saudou, 2005). Repressor element 1-silencing transcription factor (REST) has the capacity to modulate the epigenome and transcriptome (Buckley et al., 2010). HTT blocks REST-mediated transcriptional inhibition through the cytoplasmic complex of HTT-associated protein 1 (HAP1). Therefore, REST can assemble the repressor complex in the nucleus in mHTT (Hwang and Zukin, 2018). PRC2 catalyzes mono-, di-, and trimethylation of H3K27, acting as a chromatin associated methyltransferase (Laugesen et al., 2019). Notably, major targets of these genes are p53 and REST, while some of which interact with the PRC2 complex. Emerging evidence indicates that some lncRNAs are closely associated with the etiology of HD.

LncRNA human accelerated region 1 (HAR1), a direct target of REST, is a region where the sequence has been obviously changed. It is a constituent of two overlapping lncRNA loci, including HAR1A and HAR1B (Waters et al., 2021). Johnson et al. (2010) found HAR1 expression was significantly lower in HD patients. Further mechanistic study revealed that HAR1 was repressed by REST by specific DNA regulatory motifs, suggesting that the roles of HAR1 in HD progression.

MEG3 is also known as gene trap locus 2 (Gtl2). LncRNA MEG3 regulates a range of aging-related neurodegenerative diseases. Inactivation of Meg3 results in a marked increase in microvascular formation and angiogenesis-promoting gene expressions in the brain (Zhou et al., 2012). MEG3 is another target of REST and is associated with PRC2 complex (Johnson, 2012; Chang et al., 2017). Chanda et al. (2018) found that after MEG3 knockout in HD cell models, mHTT aggregates were markedly reduced and endogenous Tp53 levels were down-regulated.

Taurine-upregulated gene 1 (TUG1) is actively involved in numerous physiological processes, including modulating genes at epigenetics, transcription, post-transcription, translation, and post-translation (Guo et al., 2020). Studies have demonstrated that lncRNA TUG1 is closely associated with PRC2 among HD-related lncRNAs. Since the interaction of epigenetic regulatory complex of PRC2, the altered expression of TUG1 is related to diverse molecular pathways in HD brain. Khalil et al. (2009) indicated that TUG1 regulated the cytotoxicity of mHTT through p53.

LncRNA DiGeorge syndrome critical region gene 5 (DGCR5), also known as linc00037, is a REST-regulated lncRNA in neurodegeneration (Dong et al., 2018). The downregulation of DGCR5 in HD brain implies that DGCR5 is closely associated with transcriptional regulation in the progression of HD (Johnson et al., 2009).

NEAT1 is essential for structural integrity of the nuclear paraspeckle. A reanalysis of microarray data revealed an improvement in HD patients compared to the control group (Hodges et al., 2006). Cheng et al. (2018) confirmed that NEAT1_2 detected a threefold increase in the brains of HD patients. Besides, it has been proved that NEAT1 is overexpressed of in HD model brain of transgenic mice (Chanda et al., 2018). Moreover, the roles of NEAT1 were also demonstrated in cell models of HD (Sunwoo et al., 2017).

### Roles of lncRNAs in Amyotrophic Lateral Sclerosis

Amyotrophic lateral sclerosis primarily targets motor neurons, leading to serious disability and ultimately death from respiratory failure (Hardiman et al., 2017). RBPs mainly include fused in sarcoma/translated in liposarcoma (FUS/TLS) and TAR DNA-binding domain protein 43 (TDP43), which is involved in regulating RNA metabolism (Tischbein et al., 2019). It has been revealed that the abnormal aggregation of TDP43 and FUS/TLS directly causes the misfolding of wild-type SOD1 (wtSOD1) in ALS (Pokrishevsky et al., 2012). In FALS and SALS, the most common candidate mutations are SOD1, FUS, and C9orf72 gene both (DeJesus-Hernandez et al., 2011; Ajroud-Driss and Siddique, 2015). There is mounting evidence indicates that lncRNAs exerts a vital role in ALS pathogenesis.

LncRNA NEAT1_2 has been shown to be associated with the early course of ALS (Nishimoto et al., 2013). They also explored that the interaction between NEAT1_2 and ALS-associated RBPs, and found that FUS/TLS and TDP43 were enriched in paraspeckles. Besides, Hutchinson et al. (2007) investigated markedly raised frequency of paraplaque formation in the early stage of ALS pathology, implying that NEAT1_2 could serve as the scaffold of RBPs in the ALS motor nucleus.

Postmortem ALS tissues were examined by iCHIP, and NEAT1 was found to interact with FUS/TLS and TDP43, MALAT1 with TDP43, and MEG3 with FUS (Tollervey et al., 2011; Lagier-Tourenne et al., 2012). Moreover, Biscarini et al. (2018) found that three of the identified conservative lncRNAs, including lncMN-1, lncMN-2, and Lhx1os, were affected by FUS/TLS in ALS mouse models.

## Conclusion and Perspectives

LncRNAs knowledge has changed dramatically over the past decade and is likely to continue to in the future. It has been demonstrated that lncRNAs own diverse molecular functions, such as translation, post-translation, and epigenetic modification. Massive evidence proves that lncRNAs are involved in aging-related neurodegenerative diseases. As the aging population continues to increase, aging-related neurodegenerative diseases will become a heavy burden of society. Therefore, it is essential to further understanding of mechanisms and roles of lncRNAs in these diseases. However, there exists some challenges regarding its clinical application. In addition, the functions of lncRNAs at molecular or cellular levels remain elusive. In-depth understanding of the mechanisms and networks of lncRNAs from different perspectives will provide new insights into the prevention, diagnosis and treatment of aging-related neurodegenerative diseases.

## Author Contributions

Y-QN wrote the manuscript. HX drew the figures. Y-SL conceived the idea and supervised the manuscript. All authors read and approved the final manuscript.

## Conflict of Interest

The authors declare that the research was conducted in the absence of any commercial or financial relationships that could be construed as a potential conflict of interest.

## Publisher’s Note

All claims expressed in this article are solely those of the authors and do not necessarily represent those of their affiliated organizations, or those of the publisher, the editors and the reviewers. Any product that may be evaluated in this article, or claim that may be made by its manufacturer, is not guaranteed or endorsed by the publisher.
